# Prevalence and root causes of surgical site infections at an academic trauma and burn center in Ethiopia: a cross-sectional study

**DOI:** 10.1186/s13037-019-0229-x

**Published:** 2020-01-07

**Authors:** Rahel Mezemir, Awole Seid, Teshome Gishu, Tangut Demas, Addisu Gize

**Affiliations:** 1 Department of Surgical Nursing, St. Paul’s Hospital Millennium Medical College, Addis Ababa, Ethiopia; 2 Department of Adult Health Nursing, Bahir Dar University, Bahir Dar, Ethiopia; 3 Department of Emergency Medicine and Critical Care Nursing, St. Paul’s Hospital Millennium Medical College, Addis Ababa, Ethiopia; 4 Department Microbiology, St. Paul’s Hospital Millennium Medical College, Addis Ababa, Ethiopia

**Keywords:** Prevalence, Surgical site infection, Addis Ababa, Ethiopia

## Abstract

**Background:**

Despite modern surgical techniques and the use of antibiotic prophylaxis, surgical site infection remains a burden for the patient and health system. It is a major cause of morbidity, prolonged hospital stay, and increased health costs. Thus, the main aim of this study was to determine the prevalence and root causes of surgical site infection among patients undergoing major surgery at an academic trauma and burn center in Ethiopia.

**Methods:**

A hospital based cross-sectional study was conducted on 249 patients during 6-months’ time window. Data entered in SPSS and multivariate logistic regression was employed to determine the root causes and the outcome variable.

**Results:**

The prevalence of surgical site infection was found to be 24.6% of whom 10% develop deep site, 9.2% organ spaced and the remaining 5.2% develop superficial space surgical site infection. The prevalence was high in patients who had undergone orthopedics (54.3%) and abdominal (30%) surgeries. Educational status, pre-morbid illness, duration of pre-operative and post-operative hospital stay, ASA score, and type of the wound were significantly associated with SSI at *p*-value of ≤0.05. However, no association was found with BMI and location of the wound.

**Conclusions:**

The prevalence of surgical site infection in the study population is still high. Preoperative hospital stay, pre-morbid illness, pre-operative and post-operative hospital stay, ASA score, and type of the wound were the independent predictors of surgical site infection. The duration of pre and post-operative periods should be kept to a minimum as much as possible. Patients with pre-morbid history of chronic diseases and contaminated wound require special attention to decrease the rate of occurrence of infections. In addition, longitudinal studies should be carried out to identify more risk factors.

## Background

Surgical Site Infections (SSIs) are defined as infections apparent within 30 days of an operative procedure and most often between the 5th and 10th postoperative days. However, where a prosthetic implant is used, SSIs affecting the deeper tissues may occur several months after the operation [1].

The risk of SSI is higher in developing countries relative to developed nations. SSI accounts for over 20% of all healthcare-associated infections in surgical patients. A large cross-sectional survey of healthcare-associated infections, conducted in mainland China in 2010, reported that *E. coli* (25.9%), *S. aureus* (14.3%), and *P. aeruginosa* (11.9%) were the three most common pathogens associated with SSI [2].

The Centers for Disease Control and Prevention’s (CDC’s) National Healthcare Safety Network (NHSN) further classify SSIs as superficial incisional (involving only skin or subcutaneous tissue of the incision), deep incisional (involving fascia and/or muscular layers) and organ/space (involving any part of the body opened or manipulated during the procedure, excluding skin incision, fascia, or muscle layers) [3].

Most post-operative wound infections are hospital acquired and vary from one hospital to the other and even within a given hospital, and they are associated with increased morbidity and mortality [4]. The site of infection may be limited to the suture line or may become extensive in the operative site. The infecting microorganisms vary, depending on the type and location of surgery and antimicrobials received by the patient [5].

SSIs have been reported to be one of the most common causes of nosocomial infections; accounting for 20 to 25% of all nosocomial infections particularly in Ethiopia and generally in worldwide [6, 7]*.* SSIs have been responsible for increasing cost and morbidity and mortality related to surgical operations and continue to be a major problem worldwide [8]*.* Despite modern surgical techniques and the use of antibiotic prophylaxis, SSI is one of the most common complications encountered in surgery [1]. SSIs impose a substantial clinical burden. Surgical site infections will result for socioeconomic problem as they frequently result in hospital re-admissions, expose for frequent surgeries, demanding long-term treatment, disability, and patient morbidity. Patients with SSIs are more likely to require readmission to hospital or intensive care unit (ICU) treatment, and are at higher risk of death, than those without such infections. Most of the time, it is associated with increased age, prolonged duration of hospital stay, blood transfusion and emergency surgery [9–11].

SSI places a significant burden on both the patient and health system especially in Sub Saharan Africa, where resources are limited and the average wound infection rates are 2 or 3 times higher than developed countries due to malnutrition, poor, and preoperative preparation [12]. Moreover, in developing countries where resources are limited, even basic life-saving operations, such as appendectomies and cesarean sections, are associated with high infection rates and mortality [13].

A study conducted on prevalence and predictors of surgical site infections among patients undergoing major surgery in Tanzania revealed its incidence is higher than that reported in developed countries. The overall surgical site infection rate was 26.0%, of which 86.2% were superficial SSIs and 13.8% were deep SSIs. Regarding the predictors’ pre-morbid illness, duration of operation, use of iodine alone for skin preparation, use of drain and cigarette smoking were significantly associated with SSIs [14].

In Ethiopia, the prevalence of SSI vary from hospital to hospital even from ward to ward and it ranges from 14.8–59% and also 6.5% from private hospitals up to 13.4% in public hospitals. In Addis Ababa it was reported 47% and in Gondar 39.5%. Additionally, in Jimma University Specialized Hospital, the magnitude of clean contaminated and contaminated wound was 64.8 and 35.2%, respectively [15–17].

Even though different researches were done in different part of Ethiopia, prevalence of SSI varies from hospital to hospital even from ward to ward. Thus, we are also interested to investigate to its prevalence and root causes in St Paul hospital which is one of the teaching hospitals delivering multiple services in the country. Additionally, this study will add knowledge regarding the root causes of the problem.

## Materials and methods

A hospital based cross-sectional study was conducted prospectively from May1 to October 30, 2016. A total sample of 249 patients was recruited from St. Paul’s Hospital Millennium Medical College (SPHMMC) and Addis Ababa Burn, Emergency and Trauma Hospital. More specifically, it was conducted in the surgical, orthopedic and maternity wards of these two affiliate hospitals. SPHMMC has currently 700 beds with an annual average of 200,000 patients and has a catchment population of more than 5 million. It is a semi-independent institution under SPHMMC, providing comprehensive emergency care in emergency medicine, critical care, orthopedics, neurosurgery and forensic medical services. The hospital has a capacity of 250 beds and can serve 50–60 emergency patients at a time with an additional 12 ICU beds.

All patients who underwent major surgical procedures with a visible incision at the respective study hospitals constitute the source population, and patients examined during the study period were considered the study population. Major surgery is defined as any invasive operative procedure in which a more extensive resection is performed and involves a risk to the life of the patient.

The sample size was computed using the single proportion population formula. By considering a maximum 17.9% estimated prevalence done in Addis Ababa, precision (d = 0.05) and taking 95% confidence interval, it was estimated as 226. By adding 10% non-response rate, the final sample size was calculated as 22 + 226 = 249.

A proportional sample size was taken from General surgery (n_1_ = 89), Orthopedic (n_2_ = 80), Gynecology and Obstetrics (n_3_ = 50) and ENT (n_4_ = 30). Additionally, taking a list of post-operative patients as a sampling frame, systematic random sampling technique with k = 4 were employed to select these study subjects.

A diagnosis of a deep incisional SSI was confirmed by a surgeon or attending physician. The Centers for Diseases Control and Prevention (CDC) and National Nosocomial Infection Surveillance (NNIS) system criteria and method were used for identifying and diagnosing SSI.

Patients who showed signs and symptoms of infection within the first 48 h of admission were excluded from the study. Data was collected from post-operative patients who stayed for 48 h after surgery and before discharge. The denominator used to determine the rate was all moribund patients who are not expected to survive without the operation and underwent major surgery.

Each eligible patient was followed from the time of admission until time of discharge using a well-structured questionnaire. The data collection tool captured socio-demographic data and relevant clinical characteristics related to SSIs. Seven BSc nurses and two general practitioners (GPs) were recruited as data collectors and supervisors, respectively.

After data collection, each questionnaire was checked for completeness. Data was coded, cleaned, and analyzed using SPSS version 20. Logistic regression was used in order to test association between independent and dependent variables. Odds Ratio, 95% CI *P*-value < 0.05 was used to test associations.

Ethical clearance was obtained from the review board of SPHMMC. After the purpose and objective of the study were explained, verbal consent was obtained from each study subject and confidentiality was maintained. Participants were also informed that participation was on a voluntary basis.

## Result

A total of 249 admitted patients for major surgical procedures were studied and the response rate was 100%.

### Socio-demographic characteristics

Of the 249 samples, 40.2% were males and the rest (59.8%) were females. The mean age of study participants was 25 years (±SD 1.01) and 30.9% of the respondents were in the age range of 25–34 years. One hundred forty eight (59.4%) patients come from out of Addis Ababa, 67.5% Orthodox in religion, and 69.9% of participants were married. A substantial proportion (71.5%) had a BMI of 18-25 k.g/m^2^ which is normal weight. The educational status of respondents showed 38.6% completed primary school**.**

Concerning the length of post-operative admission, the range varied between below 3 up to 60 days. A majority of post-operated patients (34%) stayed in the hospital for 3–8 days.

Regarding other clinical variables, 51(25.4%) of infected patients had ASA score of I, and 26.1% of major surgeries were performed using general anesthesia. Most surgeries lasted less than 2 h. Of patients who stayed above 4 h pre-operatively, 38.1% develop SSIs (Table 1).

**Table 1:** Socio-demographic characteristics and clinical variables of respondents who developed surgical site infection after surgery, 2016, Addis Ababa, Ethiopia

Characteristics		SSI Infection status		
		Infected *N* (%)	Non-infected *N* (%)	Total
Age (years)	18–24	7 (14.3%)	42 (85.7%)	49 (100%)
	24–34	15 (19.5%)	62 (80.5%)	77 (100%)
	35–60	23 (30.3)	53 (69.7%)	76 (100%)
	> 60	16 (34%)	31 (66%)	47 (100%)
Sex	Male	30 (30%)	70 (70%)	100 (100%)
	Female	31 (20.8)	118 (79.2)	149 (100%)
Religion	Orthodox Christian	42 (25%)	126 (75%)	168 (100%)
	Muslim	13 (26%)	37 (74%)	50 (100%)
	Protestant	6 (19%)	25 (80.6%)	31 (100%)
Residence	Addis Ababa	27 (26.7%)	74 (73.3%)	148 (100%)
	Out of Addis Ababa	34 (23%)	114 (77%)	101 (100%)
	Single	16 (27.1)	43 (72.9%)	59 (100%)
	Married	42 (24.1)	132 (75.9%)	174 (100%)
Marital status	Divorced	2 (28.6%)	5 (71.4%)	7 (100%)
	Widowed	1 (11.1%)	8 (88.9%)	9 (100%)
	Illiterate	20 (37%)	34 (63%)	54 (100%)
Education	Primary school	19 (19.8)	77 (80.2%)	96 (100%)
	Secondary school	17 (25%)	51 (75%)	68 (100%)
	Higher Education	5 (16.1%)	26 (83.9%)	31 (100%)
	Under weight(< 18)	10 (52.6%)	9 (47.4%)	19 (100%)
BMI (kg/m^2^)	normal weight(18–25)	40 (22.5%)	138 (77.5%)	178 (100%)
	Overweight (26–29)	11 (21.2%)	41 (78.5%)	52 (100%)
	I	51 (25.4%)	150 (74.6%)	201 (100%)
ASA scoring	II	6 (14.3%)	36 (85.7%)	42 (100%)
	III	4 (66,7%)	2 (33.3%)	6 (100%)
Type of Anesthesia	General anesthesia	46 (26.1%)	130 (73.9%)	176 (100%)
	Spinal anesthesia	15 (20.5%)	58 (79.5%)	73 (100%)
	< 2 h	25 (20.9%)	95 (79.2%)	120 (100%)
Intra-operation duration	1-2 h	24 (25.5%)	70 (74.5%)	94 (100%)
	> 2 h	12 (34.3%)	23 (65.7%)	35 (100%)
Pre-operative hospital stay	< 2 h	30 (19%)	128 (81%)	158 (100%)
	2 to 4 h	15 (30.6%)	34 (69.4%)	49 (100%)
	> 4 h	16 (38.1%)	26 (61.4%)	42 (100%)
Wound Classification	Clean	0	125 (100%)	125 (100%)
	Clean-contaminated	48 (43.6%)	62 (56.4%)	110 (100%)
	Contaminated	7 (50%)	7 (50%)	14 (100%)

### Prevalence of surgical site infection and other clinical variables

In this study the overall prevalence of surgical site infection was found to be 24.5%. Moreover, the prevalence of surgical site infection was high in orthopedics and abdominal surgery which accounts 54.3 and 30% respectively and least documented in bladder surgery and cholelithiasis.

Most of the operations were elective (56.6%) and the rest (43.4%) were emergencies. Two hundred (80.3%) respondents had no pre-morbid conditions.

Regarding the duration of operation, it ranged from 0 to 2 h. The SSI rate in patients with duration of operation < 1 h was 20.8% as compared to 34.3% in above ≥2 h.

The proportion of SSI based on location of wound showed wound on back 1(16.7%), abdomen 24 (14.9%), neck and head 9 (34.6%) and leg 14(63.6%). Moreover, 198(79.5%) of participants took antibiotics before surgery.

A significant number of patients developed deep surgical site infection (10%), 9.2% developed organ spaced, and the least one 5.2% developed superficial space SSI.

### Factors associated with surgical site infection

Multivariate logistic regression analysis results showed educational status, pre-morbid illness, pre-operative and post-operative hospital stay, ASA score, and the type of wound were found to be significantly associated with SSI at *p*-value of ≤0.05.

In this study, respondents with higher education were 0.1 times [AOR: 0.1(0.03–0.91, *p* = 0.041)] less likely to predispose to SSI as compared to illiterates. On the other hand, patients who stayed > 4 h pre-operatively were 6 times more likely to develop surgical site infection as compared to < 2 h [AOR: 6 (1.5–27.9, *p* = 0.012)].

Regarding the length of post-operative stay after surgery, participants admitted for 7–14 and > 14 days were 5 times more likely to acquire surgical SSI than < 7 days [AOR: 4.3(1.11–16.10), *P* = 0.035], [AOR: 5(2.04–101.12, *p* = 0.007)] respectively.

Concerning the surgical site wound category, the clean-contaminated wound category was about 6 times [AOR: 6(5.39–35.94), *p* = 0.000] more likely to increase the risk of SSI as compared to a clean wound.

American Society of Anesthesiology (ASA) Class I patients were 0.3 times less likely to develop surgical site infection than patients with severe systemic disease (Class III) ([AOR: 0.03(0.07–1.26)] (Table 2).

**Table 2:** Multivariate analysis of factors associated with SSI in 2016, Addis Ababa, Ethiopia

Characteristic	Patients with SSI	Patients without SSI	AOR (95% CI)	P-value
Educational Status				
Illiterate	20 (37%)	34 (63%)	1	
Grade 1-8th	19 (19.8)	77 (80.2%)		
Grade 9-12th	17 (25%)	51 (75%)		
College/university	5 (16.1%)	26 (83.9%)	0.1 (0.03–0.913)	0.041
Pre-operative hospital stay				
< 2 h	30 (19%)	128 (81%)	1	
2-4 h	15 (30.6%)	34 (69.4%)		
> 4 h	16 (38.1%)	26 (61.4%)	6 (1.5–27.90)	0.012
Post-operative hospital stay				
< 7 days	34 (55.7%)	169 (89.9%)	1	
7–14 days	14 (23%)	13 (6.9%)	4.3 (1.11–16.10)	0.035
> 14 days	13 (21.3%)	6 (3.2%)	5 (2.04–101.12)	0.007
ASA score				
III	4 (66.7%)	2 (33.3%)	1	
II	6 (14.3%)	36 (85.7%)		
I	51 (25.4%)	150 (74.6%)	0.3 (0.07–1.26)	0.026
Pre-morbid illness				
Diabetes Mellitus	5 (62.5%)	3 (37.5%)	1	
Hypertension	3 (10.7%)	25 (89.3%)	0.03 (0.001–0.74)	0.032
HIV	0	4 (100%)		
No disease	50 (25%)	150 (75%)	0.002 (0.00–0.13)	0.003
Wound classification				
Clean	0	125 (100%)	1	
Clean-contaminated	48 (43.6%)	62 (56.4%)	5 (4.80–93.95)	< 0.001
Contaminated	7 (50%)	7 (50%)	6 (5.39–35.94)	< 0.001

## Discussion

In the present investigation, SSI was observed in 24.6% of patients. Even though many hospitals in Ethiopia are planning to have zero SSI, it has continued to be a drawback of quality of surgical care. This finding was lower than the study conducted in Ethiopia at Tikur Anbessa Hospital, Addis Ababa (47 to 59%) [15, 16]. The previous study was conducted far earlier, so there might be different improvements today. Similar findings were found in Tanzania 19.4%, Albania 24.3%, Norway, 28%. However, the prevalence is higher than studies conducted in other hospitals of Ethiopia, such as in Felege Hiwot Referral Hospital, 10.2%, [18] and in Gondar University Teaching Hospital 3.5% [17]. The high prevalence in our study might be attributable to:

1. Inefficient infection control practice in the hospital and inadequate environmental hygiene;
2. Number of people in theatre may be increased as it is a teaching hospital;
3. Other studies evaluated only one or two wards which might underestimate their results.

A meta-analysis report of mainland China showed the average incidence of SSI was 4.5% (95% CI: 3.1–5.8) analyzed from 2001 to 2012. This far lower figure shows that health care associated infections continued to be a threat for surgical care in Ethiopia [2].

Furthermore, in this study educational status, pre-hospital stay, duration of post-operative admission, pre-morbid status, ASA score of the wound, and type of wound were significantly associated with surgical site infection. These findings are also similar in a study reporting from Felege Hiwot Referral Hospital [18], Pakistan [19], and Nigeria [20]. However, BMI, age, and sex were not associated.

Previous studies have shown that patients with pre-morbid illnesses, such as diabetes mellitus, are at a higher risk of developing SSI due to their low immunity [14]. This study also shows patients who are co-morbid with diabetes have a higher rate developing infection as compared to hypertensive patients. However, the number of HIV infected patients was not enough to conduct association tests.

The multivariate logistic regression analysis finding showed that the rate of SSI was significantly associated with ASA classification. Similar findings have been reported by other studies [21]. In this study, ASA class 1 decreased the risk for SSIs by 0.3 times as compared to ASA 3.

Moreover, surgeries performed on contaminated wounds increase the rate of infection by 6 fold, which necessitates careful assessment of the nature of wound before surgery.

The limitation of this study could be an underestimation of the prevalence of SSI because many of these infections occur after the patient has been discharged from hospital. Besides, the research is not supported with culture investigation, which is done by other studies, so that there might be underestimation or overestimation of the prevalence.

## Conclusion

The prevalence of SSI in the study area is still high and requires due attention. Consistent infection prevention practices with commitments of the higher officials and policy makers have to be implemented in the institution. Furthermore, as it is a teaching institution, the number of students in the operating theater should be minimized as much as possible to reduce the risk of infection.

Post operation hospital stay, prolonged preoperative hospital stay, pre-morbid conditions, and contaminated and dirty wound classes were the independent predictors of SSIs.

The duration of pre- and post-operative periods should be kept to the minimum as much as possible. Patients with a pre-morbid history of chronic diseases and contaminated wounds require special attention to decrease the rate of occurrence of infections.

A longitudinal study with sound methodology should be conducted to identify more risk factors.

## Data Availability

All data generated or analyzed during this study are included in this published article.

## Abbreviations

ASA: American Society of Anesthesiology
CDC: Center for Disease Control and Prevention
ICU: Intensive Care Unit
NNISS: National Nosocomial Infection Surveillance System
SSI: Surgical Site Infection
WHO: World Health Organization
